# Marginal Lands to Grow Novel Bio-Based Crops: A Plant Breeding Perspective

**DOI:** 10.3389/fpls.2020.00227

**Published:** 2020-03-03

**Authors:** Francesco Pancaldi, Luisa M. Trindade

**Affiliations:** Plant Breeding, Wageningen University & Research, Wageningen, Netherlands

**Keywords:** bio-based economy, bio-based crops, perennial lignocellulosic crops, marginal lands, plant breeding, breeding tools

## Abstract

The biomass demand to fuel a growing global bio-based economy is expected to tremendously increase over the next decades, and projections indicate that dedicated biomass crops will satisfy a large portion of it. The establishment of dedicated biomass crops raises huge concerns, as they can subtract land that is required for food production, undermining food security. In this context, perennial biomass crops suitable for cultivation on marginal lands (MALs) raise attraction, as they could supply biomass without competing for land with food supply. While these crops withstand marginal conditions well, their biomass yield and quality do not ensure acceptable economic returns to farmers and cost-effective biomass conversion into bio-based products, claiming genetic improvement. However, this is constrained by the lack of genetic resources for most of these crops. Here we first review the advantages of cultivating novel perennial biomass crops on MALs, highlighting management practices to enhance the environmental and economic sustainability of these agro-systems. Subsequently, we discuss the preeminent breeding targets to improve the yield and quality of the biomass obtainable from these crops, as well as the stability of biomass production under MALs conditions. These targets include crop architecture and phenology, efficiency in the use of resources, lignocellulose composition in relation to bio-based applications, and tolerance to abiotic stresses. For each target trait, we outline optimal ideotypes, discuss the available breeding resources in the context of (orphan) biomass crops, and provide meaningful examples of genetic improvement. Finally, we discuss the available tools to breed novel perennial biomass crops. These comprise conventional breeding methods (recurrent selection and hybridization), molecular techniques to dissect the genetics of complex traits, speed up selection, and perform transgenic modification (genetic mapping, QTL and GWAS analysis, marker-assisted selection, genomic selection, transformation protocols), and novel high-throughput phenotyping platforms. Furthermore, novel tools to transfer genetic knowledge from model to orphan crops (i.e., universal markers) are also conceptualized, with the belief that their development will enhance the efficiency of plant breeding in orphan biomass crops, enabling a sustainable use of MALs for biomass provision.

## Introduction

A global bio-based economy where building-block materials, chemicals and energy are derived from biological biomass could significantly mitigate main environmental and social problems of our fossil-based society, including climate change, environmental pollution, and geopolitical tensions (McCormick and Kautto, 2013; Bennich and Belyazid, 2017). To address this challenge, more than 40 governments worldwide have explicitly set up strategies to transit toward bio-based economic systems (Dietz et al., 2018), and projections indicate that the biomass demand to sustain bio-based production chains will amount to 6.7–13.4 Bln tons year^–1^ in 2050, with an increase of 198–396% compared to 2011 levels (3.4 t year^–1^) (Piotrowski et al., 2015). Within the range of biomass sources to sustain such demand, dedicated herbaceous and woody crops have and will keep a prominent position (Piotrowski et al., 2015; OECD,, 2017). This raises concerns, as the allocation of agricultural land to biomass production conflicts with the cultivation of food crops (Dauber et al., 2012; Gelfand et al., 2013), hampering food security, destabilizing food prices, and constraining the access to food, especially to poor rural communities (Mol, 2010; Ribeiro, 2013).

To avoid conflict with food production, dedicated bio-based crops could be grown on marginal lands (MALs) (Samson and Girouard, 1998; Tilman et al., 2006, 2009; Fritsche et al., 2010; Gelfand et al., 2013; Post et al., 2013; Zhu et al., 2016; Carlsson et al., 2017; Mehmood et al., 2017), which are areas with marginal agronomic and economic potential for cultivation of food crops and currently not used by agriculture (Peterson and Galbraith, 1932; Dauber et al., 2012; Post et al., 2013; Shortall, 2013; Schmidt et al., 2015; Mehmood et al., 2017). Typical examples include abandoned lands [no longer used due to relocation of agriculture (Campbell et al., 2008)], degraded lands [no longer productive due to an intensive and unsustainable use (Daily, 1995; Campbell et al., 2008; Dauber et al., 2012)], or waste lands [with physical or environmental constraints to agriculture (Cai et al., 2010)]. MALs comprise 247–729 Mha worldwide (Hoogwijk et al., 2003; Tilman et al., 2006; Smeets et al., 2007; Campbell et al., 2008; Field et al., 2008; Haberl et al., 2010, 2011; Nijsen et al., 2012), thus retaining a great potential for growing dedicated biomass feedstocks. In fact, considering an average biomass yield of 7.9 t ha^–1^ from cultivation of herbaceous and woody biomass species on MALs (Nijsen et al., 2012), such acreages would be enough to supply 28–85% and 14–42% of the total biomass demand in 2050 under the 6.7 and the 13.4 Bln t scenarios, respectively.

To successfully grow bio-based crops on MALs it is critical to offset the factors that determine the marginality of these areas (Fritsche et al., 2010; Dauber et al., 2012). Most of these factors are physical and agronomic, such as adverse land morphology (e.g., steep slopes), unfavorable soil conditions (e.g., low fertility, salinity, acidity, erodibility, poor drainage, contamination by heavy metals), or hostile climate (e.g., recurrent droughts and floods, extreme temperatures) (Dauber et al., 2012; Gelfand et al., 2013; Jones et al., 2014; Meyfroidt et al., 2016; Smaliychuk et al., 2016). Others are socio-economic, as the lack of adequate transport infrastructures, undefined land ownership, or low mechanization levels and negative demographic conditions (e.g., low population density and educational level) in the regions where MALs are located (Fritsche et al., 2010; Dauber et al., 2012; Meyfroidt et al., 2016; Smaliychuk et al., 2016). Offsetting marginality through the planting of bioenergy crops requires therefore the design of cropping systems that match the site-specific conditions of each MAL at all the levels of the agricultural practice.

At the crop and agronomic level, this means adopting crops and practices that can sustain large yields of high-quality biomass (see section “Biomass Quality” for the definition of “biomass quality” in the context of biomass production on MALs) under unfavorable conditions, minimize input requirements and promote an ecological restoration of MALs (Zegada-Lizarazu et al., 2010; Dauber et al., 2012; Allwright and Taylor, 2016). Currently available crops hardly match these conditions, as they have been bred for centuries toward different targets than the production of bio-based commodities and the cultivation under MALs conditions (Trindade et al., 2010). As a result, their cultivation on MALs could even promote soil erosion and runoff of fertilizers and pesticides (Campbell et al., 2008; National Research Council,, 2008), and would likely require high external inputs to sustain optimal biomass yields (de Vries et al., 2010; Parenti et al., 2018). An array of new crops tailored to marginal environments should therefore be developed, with a central role for plant breeding. This review aims at elucidating which crops are best to be sustainably grown on MALs, which traits should be prioritized in their improvement, and which tools are effective to advance those crops and traits.

## Perennial Lignocellulosic Crops: a Promising Cropping System for Biomass Production on Marginal Lands

Several studies indicate mixtures of perennial lignocellulosic biomass crops grown under low-input agriculture as suitable cropping systems for biomass production on MALs (Tilman et al., 2006, 2009; Dauber et al., 2012; Gelfand et al., 2013; Carlsson et al., 2017; Mehmood et al., 2017; Robertson et al., 2017). In this paragraph, we discuss why such systems fit well MALs conditions, and which factors are critical to ensure that their advantages are effectively delivered.

### Advantages of Cultivating Mixed Perennial Lignocellulosic Crops on Marginal Lands

Mixed perennial biomass crops (MPBCs) can couple the provision of lignocellulosic biomass with the improvement or the restoration of the ecological services of MALs at all the ecosystem levels.

Regarding soil properties, MPBCs enhance soil structure and reduce erosion (Blanco-Canqui et al., 2014; Cosentino et al., 2015; Blanco-Canqui, 2016; LeDuc et al., 2017; Fernando et al., 2018), owing to a dense and prolonged soil coverage, as well as deep and branched roots (Fernando et al., 2018), which hold large amounts of water and nutrients (Blanco-Canqui, 2016; Fernando et al., 2018). This, together with the high resource use efficiency of these crops (van der Weijde et al., 2013; Lewandowski, 2016; Carlsson et al., 2017) and their low or null fertilization requirements (Tilman et al., 2006; Robertson et al., 2017; Fernando et al., 2018), determines the very low levels of nutrient leaching (especially nitrogen – N) observed in MPBCs (Glover et al., 2010; Pérez-Suárez et al., 2014; LeDuc et al., 2017; Robertson et al., 2017; Fernando et al., 2018). For example, McIsaac et al. (2010) reported that, over a 4-years comparison, unfertilized miscanthus and switchgrass leached on average 3.0 and 1.4 kg N ha^–1^ year^–1^, respectively, far lower than a conventional maize-soybean rotation (40.4 kg N ha^–1^ year^–1^). Similar conclusions have been reached in several other studies focused on different biomass perennials (e.g., poplar, willow, switchgrass, grass-legume), as thoroughly reviewed by Robertson et al. (2017). MPBCs improve also soil organic carbon (SOC) stocks (Anderson-Teixeira et al., 2009; Glover et al., 2010; Pérez-Suárez et al., 2014; LeDuc et al., 2017; Robertson et al., 2017; Fernando et al., 2018). In this regard, Chimento et al. (2016) showed that, over a 6-years trial, perennial herbaceous (giant reed, miscanthus, switchgrass) and woody (poplar, black locust, willow) species accumulated on average 45% higher SOC than continuous tilled corn in the soil portion interested by roots. This large carbon (C) sequestration results from the continuous ground coverage, the low soil disturbance, and the large rooting systems of MPBCs (Carlsson et al., 2017), and is maximized when MPBCs are established over MALs that do not store large C stocks in their soils or natural vegetation (Tilman et al., 2009; Gelfand et al., 2013). Therefore, such “low-C MALs” should be prioritized for allocation to biomass production over areas that are naturally evolving to C-rich ecosystems (e.g., forests or wetlands) (Tilman et al., 2009; Robertson et al., 2017). An incautious use of C-rich MALs for biomass production could in fact even generate a large C debt (Schulze et al., 2012; Robertson et al., 2017), that could require decades or centuries for repaying (Fargione et al., 2008; Gibbs et al., 2008; Gelfand et al., 2011).

MPBCs can also contribute to preserve water resources. Their absolute water needs per ha generally equal – or even outweigh – annual crops as maize, wheat, or sorghum (Robertson et al., 2011c, 2017; van der Weijde et al., 2013; Hamilton et al., 2015; Fernando et al., 2018), since their large biomass production and prolonged growing season implicate high evapotranspiration rates (Hamilton et al., 2015; Robertson et al., 2017). However, water use efficiencies (WUEs) of MPBCs are generally high (especially for C4 species) (Robertson et al., 2011c; van der Weijde et al., 2013; Zeri et al., 2013), which makes them best candidates for a water-effective production of biomass (Mehmood et al., 2017). This is especially true considering that several biomass perennials are drought tolerant (Mehmood et al., 2017), can be irrigated using wastewaters (Barbosa et al., 2015), and can suffice their water needs with seasonal rainfalls in temperate climates (Robertson et al., 2017). In addition, MPBCs improve water quality and water management, as their extensive roots, prolonged soil coverage, and positive effect on soil structure and porosity promote water penetration into soils (Blanco-Canqui, 2016; Fernando et al., 2018), minimizing water runoff and soil erosion (Blanco-Canqui, 2016; Acharya and Blanco-Canqui, 2018). Furthermore, the low leaching fluxes and little agrochemical needs of MPBCs minimize water pollution (Blanco-Canqui, 2016; Acharya and Blanco-Canqui, 2018). Finally, several perennial crops (e.g., willow, giant reed, miscanthus) can sequester contaminants (e.g., Cd, Pb, Zn, Cu) and pollutants from soils and water (Bandarra, 2013; Boléo et al., 2015; Costa et al., 2016), being therefore good options to remediate contaminated MALs and depurate polluted water streams (Barbosa et al., 2015; Costa et al., 2016; Acharya and Blanco-Canqui, 2018).

Biodiversity is also improved under the cultivation of MPBCs. Werling et al. (2014) showed that these cropping systems display a much wider diversity than monocultures of annual biomass crops as maize with respect to several taxonomic groups (microorganisms, arthropods, birds, and plants). These results are in line with several other studies that compared the diversity within specific biological clades in annual and perennial bioenergy crops, as Meehan et al. (2010) and Robertson et al. (2011b) (birds), Gardiner et al. (2010); Bennett et al. (2014), and Haughton et al. (2016) (insect communities), Levine et al. (2011) (methanotrophic bacteria), or Haughton et al. (2016) (plants). In addition, the specific change in land use from degraded MALs to MPBCs enhances biodiversity (Dauber et al., 2010; Meehan et al., 2010; Robertson et al., 2011a; Chauvat et al., 2014), and the enhancement is larger as the species diversity of the new cropping system is wider (Dale et al., 2010; Landis et al., 2018). The increase in biodiversity under biomass perennials boosts also fundamental ecosystem services for agriculture, as pollination (Bennett and Isaacs, 2014; Carlsson et al., 2017) or pest suppression (Werling et al., 2011; Carlsson et al., 2017), which allows for a reduced use of agrochemicals without compromising yields (Carlsson et al., 2017). Moreover, these benefits are even extended to neighboring annual croplands, where pollination, pest suppression and yields can increase up to ∼25%, thanks to the enhanced ecosystem functions of nearby MPBCs (Liere et al., 2015).

To conclude, MPBCs can also restore an economic value and an agrarian revenue to degraded lands, improving rural development. In developed countries, their cultivation contributes to diversify farm income from arable and grass lands, and offer new occupational perspectives to older or part-time farmers (Valentine et al., 2012). Alternatively, novel bioenergy production chains based on biomass from MALs can create employment opportunities in developing countries, as well as offer access to novel, clean, energy sources, which improves living and economic standards of local communities (Valentine et al., 2012).

### Steps to Establish Mixed Perennial Biomass Crops on MALs

To effectively deliver the benefits promised by MPBCs, the species and the management adopted should reflect the site-specific conditions of each target MAL (Zegada-Lizarazu et al., 2010; Blanco-Canqui, 2016; Robertson et al., 2017). Therefore, the preference should go for a wide array of dedicated and locally adapted biomass perennials, each of which fitted to specific ecological niches, and globally suitable for diverse environmental scenarios (Jones et al., 2015; Robertson et al., 2017). Table 1 reports a list of possible crops. These species typically withstand well the poor MALs conditions, showing constitutive resistance to several abiotic stresses, displaying high resource use efficiencies, and requiring low inputs (Zegada-Lizarazu et al., 2010; Dauber et al., 2012; Mehmood et al., 2017). However, most of them are novel or orphan crops, which did not undergo genetic improvement so far, especially with respect to biomass-related traits (Zegada-Lizarazu et al., 2010; Dauber et al., 2012; Jones et al., 2015; Zhu et al., 2016). As a consequence, their biomass yield and quality are highly variable (Zegada-Lizarazu et al., 2010), and often considerably lower than their genetic potential (Allwright and Taylor, 2016). This is critical, as the economic viability of cultivating MPBCs on MALs largely depends on their capacity of not only surviving structural and contingent suboptimal and low-input growing conditions, but of also producing, under those scenarios, large and high-quality biomass yields (Dauber et al., 2012; Blanco-Canqui, 2016). On the one hand, high-yielding and robust varieties significantly increase farmers’ willingness of cultivating biomass perennials on MALs, by decreasing the opportunity cost of land and increasing profits (Soldatos, 2015). On the other hand, the provision of feedstocks with optimized lignocellulose composition in relation to the intended bio-based end uses (e.g., fermentation into biofuels, extraction of plant chemicals, or transformation into biomaterials as textile fibers) is critical to increase the profitability and competitiveness of the industrial use of plant biomass (Trindade et al., 2010). To enable the cultivation of MPBCs on MALs, it will be thus essential to breed varieties that couple robustness with optimal yields (Jones et al., 2015).

**TABLE 1:** Promising perennial lignocellulosic crops for cultivation on MALs.

**Crop**	**Habit**	**Biomass yield (tons ha^–1^ y^–1^)**	**Available genetic resources to support breeding**	**References**
Miscanthus (*Miscanthus* spp.)	Herbaceous	15–19	Genome sequence; genetic maps; QTLs for biomass quality, agronomic performance, plant morphology	Atienza et al., 2002, 2003a,b; Kim et al., 2012; Ma et al., 2012; Swaminathan et al., 2012; Gabrielle et al., 2014; Liu et al., 2014, 2016; Gifford et al., 2015; van der Weijde, 2016, van der Weijde et al., 2017b; Xue et al., 2016; Baute et al., 2018
Switchgrass (*Panicum virgatum* L.)	Herbaceous	1-22	Genetic maps, EST databases; SNP arrays; genome samplings; BAC libraries; analyses of natural variation; QTLs for biomass, yield, plant height, flowering time, reproductive maturity	Casler et al., 2011; Gabrielle et al., 2014; Dong et al., 2015; Lowry et al., 2015; O Di Nasso et al., 2015; Serba et al., 2015; Bartley et al., 2016; Bahri et al., 2018; Baute et al., 2018; Tornqvist et al., 2018
Giant reed (*Arundo donax*)	Herbaceous	7-61	Mutant and clone collections; leaf and shoot transcriptomic sequences; studies on genetic diversity (AFLP, RAPD, and microsatellites markers)	Khudamrongsawat et al., 2004; Zegada-Lizarazu et al., 2010; Pilu et al., 2014; Barrero et al., 2015; Canavan et al., 2017; Evangelistella et al., 2017; Malone et al., 2017; Valli et al., 2017
Reed canarygrass (*Phalaris arundinacea* L.)	Herbaceous	15	N.A.	Pocienë et al., 2013; Lord, 2015
Virginia mallow (*Sida hermaphrodita*)	Herbaceous	9-20	N.A.	Borkowska et al., 2009; Franzaring et al., 2014
Cardoon (*Cynara cardunculus* L.)	Herbaceous	7-15	Genetic maps; marker arrays; QTLs for yield, biomass production, earliness	Acquadro et al., 2005; Lanteri et al., 2006, 2012; Angelini et al., 2009; Portis et al., 2009, 2012, 2014; Sonnante et al., 2011; Scaglione et al., 2012; Mauromicale et al., 2014; Martin et al., 2015; Francaviglia et al., 2016
Agave (*Agave* spp.)	Herbaceous	1-34	RAPD marker array; transcriptomic sequences	Vega et al., 2001; Davis et al., 2011; Gross et al., 2013
Tall wheatgrass (*Thino pyrum ponticum*)	Herbaceous	3-11	N.A.	Wang et al., 2010, 2014; Pearson et al., 2015
Bamboo (*Bambusa balcooa*)	Herbaceous	40-50	N.A.	Kuehl, 2015
Cup plant (*Silphium perfoliatum* L.)	Herbaceous	7-13	N.A.	Šiaudinis et al., 2015
Common reed (*Phragmites australis*)	Herbaceous	10-18	N.A.	Hansson and Fredriksson, 2004; Baute et al., 2018
Spanish broom (*Spartium junceum* L.)	Herbaceous	18	Studies on the genetics underneath adaptive traits for growth on steep soils	Angelini et al., 2000; Scippa et al., 2005; Di Michele et al., 2006
Nettle (*Urtica dioica* L.)	Herbaceous	6-15	Expression study of *SUS* genes related to fiber synthesis and quality	Hartl and Vogl, 2002; Bacci et al., 2009; Backes et al., 2018
Poplar (*Populus* spp.)	Woody tree	7-28	Genome sequence; genetic maps based on different types of molecular markers; QTLs for plant growth, morphology, phenology, root growth, biomass yield, cell wall quality, wood composition	Bradshaw et al., 1994; Bradshaw and Stettler, 1995; Wu, 1998; Wu et al., 2000; Cervera et al., 2001; Yin et al., 2004; Zhang et al., 2004, 2006, 2009; Tuskan et al., 2006; Gaudet et al., 2008; Rae et al., 2008; Pakull et al., 2009; Ranjan et al., 2010; Zegada-Lizarazu et al., 2018
Willow (*Salix* spp.)	Woody tree	5-30	Genome sequenced; genetic maps; QTLs for growth, biomass yield, cold tolerance, drought tolerance, plant phenology, enzymatic saccharification	Hanley et al., 2002; Tsarouhas et al., 2002, 2004; Ronnberg-Wastljung et al., 2003; Rönnberg-Wästljung et al., 2005; Hanley et al., 2006; Aylott et al., 2008; Brereton et al., 2010; Zegada-Lizarazu et al., 2010; Berlin et al., 2014; Gabrielle et al., 2014; Talukder and Saha, 2017
Black locust (*Robinia pseudacacia*)	Woody tree	10	N.A.	Amaducci et al., 2017
Eucalyptus (*Eucalyptus* spp.)	Woody tree	10-26	Genome sequenced; transcriptomic sequences; genetic maps; QTLs for plant growth, wood quality, lignin biosynthesis, vegetative propagation	Grattapaglia and Sederoff, 1994; Grattapaglia et al., 1995, 1996; Marques et al., 1998, 2002; Thamarus et al., 2002; Kirst et al., 2004; Brondani et al., 2006; Freeman et al., 2009; Myburg et al., 2011, 2014; Zegada-Lizarazu et al., 2018
Siberian elm (*Ulmus pumila* L.)	Woody tree	5-19	Chloroplast genome sequenced; breeding efforts targeting resistance to Dutch Elm Disease and wood quality	Santini et al., 2004; Geyer et al., 2007; Lamers and Khamzina, 2008; Perez et al., 2014; Zuo et al., 2017
Wild tobacco (*Nicotiana glauca* Graham)	Woody tree	3-9	Expression study under drought stress; investigation of the genetic basis of metal tolerance	Curt and Fernández, 1990; Smart et al., 2001; Shingu et al., 2005

## Target Traits and Genetic Resources to Tailor Novel Biomass Crops to MALs

The improvement of biomass yield and quality in novel species for MALs depends on the enhancement of both biomass yield and quality *per se*, and biomass yield and quality *stability* under variable abiotic conditions. This paragraph illustrates which traits are critical to achieve these equally important breeding goals, which plant ideotypes are effective to guide the improvement of such traits, and which breeding resources are currently available.

### Biomass Yield

Biomass yield is a highly complex trait, overall determined by three factors: the efficiency of light interception by the crop canopy, the efficiency of light conversion into biomass, and the efficiency of biomass partitioning into target harvestable plant components (Monteith, 1972, 1977). Thus, the enhancement of biomass yield encompasses breeding for all the morphological, physiological and phenological traits that are at the basis of these three factors (Jones et al., 2015). In addition, the characteristics of vegetative tissues should deserve particular attention, as in biomass crops they not only operate light interception and conversion into biomass, but are also the main harvest product.

#### Efficiency of Light Interception

**FIGURE 1:**
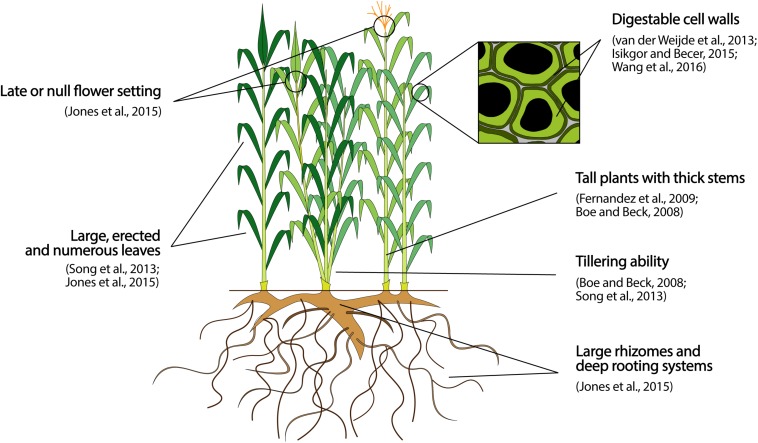
Preeminent architectural, phenological, and quality target traits to breed perennial lignocellulosic biomass crops for biomass production on MALs. The traits reported represent an ideotype to guide breeding activities, and the effective magnitudes of improvement attainable with respect to each trait can vary extensively depending on the species object of a breeding program.

The efficiency of light interception depends on plant architecture and duration and timing of crop growth (Jones et al., 2015). Plant architecture refers to crop growth habit (i.e., plant height and branching pattern) and leaves characteristics (i.e., number, size, shape, distribution and orientation) (Reinhardt and Kuhlemeier, 2002; Hollender and Dardick, 2015; Pan et al., 2017), and affects biomass yield by determining the plant density and biomass volume achievable per land unit, as well as the degree of soil coverage and photosynthetic area of the canopy. Tall plants, high tiller/stem number and density, thick stems, and upright, large and numerous leaves are all architectural characters positively correlated with biomass yield and light penetration and interception by the canopy in herbaceous biomass crops, as shown in Figure 1 (Boe and Beck, 2008; Fernandez et al., 2009; Song et al., 2013). In woody species, vertical growth habit and production of sylleptic branches are also important to attain high plant densities and increase biomass yield per hectare (Rae et al., 2004; Marron et al., 2006; Dubouzet et al., 2013). For most of these traits, variability has been observed for crops suitable for MALs, as shown by e.g., Das et al. (2004) (switchgrass), Robson et al. (2013) (miscanthus), Cosentino et al. (2006) (giant reed), or Orlovic et al. (1998), and Rae et al. (2004) (poplar). In addition, QTLs underlying architectural traits have also been mapped in two of the most studied novel biomass species [miscanthus (Atienza et al., 2003a; Gifford et al., 2015; Ge et al., 2018), and switchgrass (Serba et al., 2015)], as well as in model biomass crops as maize (e.g., Pan et al., 2017) or poplar (Bradshaw and Stettler, 1995; Zhang et al., 2006; Rae et al., 2008). These studies highlight the high genetic complexity of plant architecture, as particularly exemplified by Pan et al. (2017), who found nearly 800 QTLs associated with ten critical maize architectural traits, including plant height, number and length of branches, and leaf number, size, and orientation. Such a complexity hampers breeding for plant architecture in novel crops for which breeding tools are largely missing. Nevertheless, major-effect architectural loci have also been identified in model energy crops as maize, sorghum, or poplar, and in the model plant Arabidopsis (see Wang and Li, 2006; Fernandez et al., 2009; and Teichmann and Muhr, 2015 for detailed reviews). These loci could potentially be targeted in candidate gene approaches, by mining eventual homologous in novel biomass crops, screening allelic diversity, and selecting or introgressing favorable alleles. Preeminent examples are the *LG1* and *LG2* loci of maize, that, once mutated, induce upright leaves lacking of ligules and auricles, allowing for higher planting densities (Tian et al., 2011). Alternatively, four *DWARF* loci (*DW1*-*DW4*) largely control internode length – and therefore plant height – in sorghum in a pure Mendelian fashion (Hilley et al., 2016; Mullet, 2017).

The duration and timing of crop growth affects biomass yield by determining both the total amount of solar radiation that can be captured during crop growth, and the duration of vegetative growth prior to switching to the reproductive cycle, which typically terminates the synthesis of vegetative tissues (Fernandez et al., 2009; Jones et al., 2015). To maximize biomass yield, an ideal crop should thus display a long growing season, fully develop the canopy by the time solar radiation reaches its yearly maximum, and postpone or even avoid the setting of reproductive structures (Figure 1) (Jones et al., 2015). Achieving this ideotype implies breeding for an early leaf development, a delayed plant senescence, and a late flowering time. As a proof-of-concept, Dohleman and Long (2009) showed that the 4-weeks earlier canopy development and the 4-weeks longer canopy duration of miscanthus compared to maize largely explain the on average 60% higher biomass production of the former in US Midwest. The genetics underlying these developmental traits are complex, as a high number of genes showing pleiotropic and epistatic effects and interacting with environment are involved (see e.g., Wu et al., 2012; Gregersen et al., 2013; Bluemel et al., 2015 for reviews). Nevertheless, some critical genes can be targeted in candidate gene or transgenic approaches. An example is the *IPT* gene from *Agrobacterium tumefaciens* that, once introgressed in several plants, boosts a rate-limiting step in the cytokinin biosynthesis and delays plant senescence by sustaining high cytokinin production along the whole growing season (Gregersen et al., 2013). Genes proven to evenly affect a critical trait across several species are undoubtedly important targets to advance novel crops without the need of *de novo* investigating the genetics underlying that trait in each species. However, they are generally rare for developmental traits whose genetic architecture relies on tens of small-effect loci (Buckler et al., 2009) and has been shaped by selective forces acting in a species- and environmental-specific manner during evolution, as discussed by Gifford et al. (2015) for flowering time in miscanthus. Therefore, conventional selection of superior genotypes in target MALs is nevertheless a time- and money-saving breeding approach for traits as earliness of leaf development, delayed plant senescence and late flowering time. In this regard, variation for all of them exists in several crops, as miscanthus (Farrell et al., 2006; Jensen et al., 2011; Robson et al., 2012), or switchgrass (Van Esbroeck et al., 1997; Bhandari et al., 2010). In addition, Van Esbroeck et al. (1997) and Bhandari et al. (2010) showed that the heritability of all these traits in switchgrass is moderate to high, promising success for selection. Finally, different ecotypic groups are often encountered in species that grow over wide geographical ranges [e.g., switchgrass Evans et al. (2017) or hemp Salentijn et al. (2015)], and represent an additional resource to enhance yield. For example, when short-day hemp cultivars are cultivated at northern latitudes native of long-day genotypes, flowering time is postponed and fiber yield increases (Salentijn et al., 2015).

#### Efficiency of Light Conversion Into Biomass

The efficiency of sunlight conversion into biomass is determined by the amount of biomass energy produced relative to the total sunlight energy intercepted by a crop over a specific period of time (Zhu et al., 2010), and depends directly on crop photosynthesis. Crops with C4 photosynthesis typically display higher conversion efficiencies than C3 species, owing to a CO_2_-concentrating mechanism that prevents photorespiration by sustaining high CO_2_ leaf concentrations for optimal Rubisco activity even at low atmospheric CO_2_ levels (van der Weijde et al., 2013). Such higher photosynthetic efficiency translates into higher yield potentials of C4 relatively to C3 crops (van der Weijde et al., 2013), which make the former more suitable for cultivation on MALs. This is especially true considering that C4 plants generally outperform C3 species also in terms of nitrogen and water use efficiency, given the low levels of photosynthetic proteins in leaves and low stomatal conductance (Ghannoum et al., 2011).

Promising C4 perennials for biomass production on MALs include miscanthus, switchgrass, napiergrass or Indian grass. Moreover, research is investigating how to engineer C4 photosynthesis in C3 crops (Zhu et al., 2010; Schuler et al., 2016), which could hypothetically further enhance the biomass yield of already high-yielding C3 species suitable for MALs, as giant reed or tall wheatgrass. This appears a long-term goal though (Zhu et al., 2010), as the high complexity of C4 photosynthesis and the high number of genes involved in determining all its metabolic and physiological benefits did not allow so far to achieve satisfactory results (Schuler et al., 2016).

In addition, C4 plant species evolved as a result of convergent evolution in warm, tropical, climates (Schuler et al., 2016), and C3 plants can outperform C4 species for both biomass production and resource use efficiency at temperate latitudes (Karp and Shield, 2008; Carroll and Somerville, 2009). Where this condition applies, the intra- and inter-specific variation in photosynthetic capacity that has been nevertheless observed across several C3 species (Hikosaka, 2010; Lawson et al., 2012) represents a precious resource to improve the efficiency of light conversion in promising C3 crops for MALs. For example, Wullschleger (1993) assessed the photosynthetic capacity of 109 C3 species and found carboxylation rates in a range of 6-194 μmol m^–2^ s^–1^. This variation is thought to arise mainly from different biochemical capacities of the photosynthetic machinery (e.g., antenna complex size or photosystem II photoprotection capacity), a variable degree of CO_2_ diffusion in leaves, different rates of N supply to the photosynthetic systems, and changes in the activity of photosynthetic enzymes (Hikosaka, 2010; Zhu et al., 2010; Lawson et al., 2012). Moreover, it appears largely genetically controlled (Zhu et al., 2010; Flood et al., 2011), even if its genetic basis remains largely understudied (Flood et al., 2011; Lawson et al., 2012; Zhu et al., 2016). Recently, QTLs for photosynthetic efficiency have been identified in model species as Arabidopsis (van Rooijen et al., 2015) or tomato (De Oliveira Silva et al., 2018). However, long time will be needed before critical genetic elements are identified underneath genomic regions associated with photosynthesis variation, and the validity of such genetic elements can be extended also to orphan crops for MALs. Nonetheless, this approach could lead to the discovery of key candidate genes to improve yield of biomass crops, and should therefore be explored in further detail.

#### Efficiency of Carbon Partitioning Into Vegetative Tissues

The efficiency of C partitioning refers to the amount of fixed C invested in developing vegetative tissues over the total fixed C (Zhu et al., 2010). In biomass perennials, the seasonal production of vegetative tissues is initially fueled by the C stored into roots, until the new canopy develops sufficient photosynthetic capacity to take on this role. Therefore, two main crop characteristics are critical to maximize the biomass synthesis from a C partitioning perspective (Jones et al., 2015). Firstly, a preferential allocation of fixed C to the production of vegetative tissues over other C sinks. Secondly, a rooting system that develops its full size soon after crop establishment and capable of storing large C stocks, as displayed in Figure 1.

The preferential allocation of fixed C to the production of vegetative tissues can be improved through indirect selection for architectural and phenological traits correlated with biomass yield. In this sense, plant height and late flowering time are key, as they typically correlate with each other and with biomass yield, as shown in maize (Lübberstedt et al., 1997), sorghum (Murray et al., 2008; Ritter et al., 2008), switchgrass (Bhandari et al., 2010, 2011), or miscanthus (Gifford et al., 2015). Tall plants display a relaxed C demand by sinks different than vegetative tissues thanks to a postponed switch to reproductive growth, which translates into higher relative C investments in biomass production and larger yields. Importantly, these associations appear genetically determined, as the co-localization of QTLs for plant height, flowering time, and biomass yield has also been observed, for example in sorghum (e.g., Lin et al., 1995; Murray et al., 2008; Ritter et al., 2008). Alternatively, C partitioning can also be improved through transgenic approaches, by altering the expression of sucrose synthases or sucrose transporters (Braun et al., 2013; Yadav et al., 2015). For example, Poovaiah et al. (2015) considerably increased plant height (+37%), biomass yield (+13.6%), and tiller number (+79%) in switchgrass by overexpressing a constitutive Sucrose Synthase (*PvSUS1*) ubiquitously present in the plant. Despite the promising results, a major drawback of these approaches is that undesired side-effects on growth or physiological traits are often encountered, which calls for a deeper understanding of the genetics underlying C allocation into a plant system perspective (Yadav et al., 2015).

Improving roots and rhizomes as determinants of C allocation in biomass crops is challenging, as studying these traits is costly, time-consuming, and technically demanding, especially in field conditions (Sartoni et al., 2015; Pierret et al., 2016). Thus, little is known on the genetics of root growth (Topp et al., 2013), especially in biomass crops. In this context, the studies on the genetic basis of rhizomatousness in sorghum using progeny from crosses of wild perennial and cultivated annual accessions (Paterson et al., 1995; Kong et al., 2015) appear particularly relevant. Following this strategy, Kong et al. (2015) mapped seven major QTLs for rhizomatousness, finding co-localizations with regions affecting tillering. The further investigation of causative genes underneath these QTLs could identify candidates to improve both rooting capacity and biomass yield components of sorghum and, possibly, other biomass perennials. In addition, novel phenotyping platforms (e.g., Nadezhdina et al., 2012; Topp et al., 2013; Sartoni et al., 2015) are expected to reduce costs and destructiveness of root phenotyping, as well as grant assessment of root growth over multi-years trials, which is needed to understand root development in a C partitioning perspective. At the moment, the most feasible strategy to breed for fast-developing and large roots is by making use of known correlations between root properties and this trait ideotype. Specifically, above-ground biomass production in MPBCs positively correlates with rooting depth (Mueller et al., 2013), which in turn affects the total C sequestration capacity of roots and rhizomes (Jones et al., 2015). Therefore, root depth appears a promising target to breed for root systems capable of storing large C stocks and sustaining abundant yields.

### Biomass Quality

MPBCs produce large amounts of lignocellulosic biomass, which represents a highly attractive material for bio-based applications, as lignocellulose contains several classes of economically interesting compounds, including biopolymers and biochemicals (Trindade et al., 2010; Isikgor and Becer, 2015). The extraction of target molecules from lignocellulose is currently based on expensive and intensive post-harvest biomass treatments aimed at both loosening the structure and fractionating the components of plant cell walls, which are by far the major constituents of lignocellulose and contain the major part of attractive compounds (van der Weijde et al., 2013; Isikgor and Becer, 2015; Lauria et al., 2015; Wang et al., 2016). For this reason, the biomass quality of MPBCs entails primarily the recalcitrance of plant cell walls to deconstruction, as feedstocks with easily destructible cell walls require milder and cheaper treatments to be processed into bio-based end-products (van der Weijde et al., 2013; Isikgor and Becer, 2015; Wang et al., 2016). Moreover, the relative content of molecules of interest within cell walls in relation to the end-use of the biomass is also a preeminent quality target in order to develop crop varieties tailored to specific bio-based production chains (Trindade et al., 2010). In this regard, Table 2 reports a list of cell wall ideotypes that can fit the needs of different possible end-uses of the lignocellulosic biomass obtainable from MPBCs grown on MALs.

**TABLE 2:** Possible end-uses of the lignocellulosic biomass obtainable from MPBCs grown on MALs and relative cell wall ideotypes to optimize biomass quality toward those applications.

**Biomass application**	**Optimal cell wall ideotype to increase the biomass quality toward target applications**	**References**
Combustion for heat or energy generation	High lignin/low cellulose and hemicelluloses relative contents to increase the caloric value of the biomass. Low amounts of mineral components to avoid ash formation and corrosion of combustion chambers upon burning.	van der Weijde et al., 2017c
Production of bioethanol	Lignin: low relative content, low degree of cross-linking with cellulose and hemicelluloses. Cellulose: high relative content, low degree of polymerization, low crystallinity index. Hemicelluloses: high relative content, low degree of branching with cellulose and lignin (largely achievable through a low rate of xylan substitutions).	van der Weijde et al., 2013; Torres et al., 2015b; da Costa et al., 2019
Production of textile fibers	Lignin: low relative content. Cellulose: high relative content, high crystallinity index, small microfibril angles. Hemicelluloses: low rate of xylan substitutions to decrease cross-linking with cellulose, lignin and pectins. Others: low rates of pectin methylation, low cross-linking with other structural cell wall components.	Ranalli, 2004; Salentijn et al., 2015
Biorefining into polymers/chemicals	Lignin: high relative content for production of aromatic molecules of nutraceutical, cosmetic, pharmaceutical, and chemical interest, otherwise low relative content; tailor relative contents of S, G and H monolignols to the desired properties of end-products. Cellulose: high relative content for production of glucose and related functionalized derivatives; low crystallinity index. Hemicelluloses: high relative content for production of C_5_ (xylose, arabinose) and C_6_ (mannose, galactose, rhamnose) sugars and related functionalized derivatives to be used as polymer backbones or pendant groups in chemical industries; altered relative abundance of the different hemicelluloses polysaccharides based on the wishes of end-users. Others: optimal design of the cross-links between structural cell wall components and of the deposition of cell wall molecules within cell walls to obtain specific properties in target molecules and/or facilitate extraction and processing.	Isikgor and Becer, 2015; da Costa et al., 2019

The general structure of cell walls is conserved across plants, consisting of cellulose fibers in a matrix of non-cellulosic polysaccharides (mainly hemicelluloses), lignin, structural proteins and mineral elements (Cosgrove, 2005; Vogel, 2008). However, the relative abundance, composition, and structure of cell wall components vary extensively across species, tissues and developmental stages (Vogel, 2008; Sarkar et al., 2009; Loque et al., 2015). Likewise, the occurrence and functionality of major cell wall synthetic genes is also conserved across plants (e.g., *CESAs* and *CSLs* as main players of cellulose and hemicellulose biosynthesis, respectively) (Vogel, 2008; Xu et al., 2009; Zhang et al., 2018), even if inter-specific differences exist also at the genetic level, which affect cell wall composition (e.g., the presence of *CSL-Fs* and *CSL-Hs* only in certain plant clades, which synthesize mixed-linkage glucans) (Vogel, 2008; Doblin et al., 2009). Taken together, these observations point out a large margin for the improvement of cell wall composition toward low-recalcitrant and purpose-made cell wall ideotypes. However, the extreme complexity of cell wall biosynthesis and regulation [∼4000 genes are thought to be involved in Arabidopsis (Wang et al., 2012)] hampers breeding efforts, especially in novel crops lacking genetic tools. In this context, dissecting the trait “cell wall quality” into its main determinants (content, composition, and structure of lignin and cell wall polysaccharides) can help to better define the goals targetable by breeding, and the available biochemical and genetic knowledge to achieve them.

#### Lignin

Lignin is the cell wall component that mostly limits lignocellulose deconstruction (Li et al., 2016). On the one hand, it cross-links with hemicelluloses forming a physical barrier that hides polysaccharides to degrading enzymes (Zhao et al., 2012; Li et al., 2016). On the other hand, it irreversibly absorbs hydrolytic enzymes, inhibiting their activity (Zhao et al., 2012; Li et al., 2016). To decrease biomass recalcitrance, an immediate strategy is to decrease the lignin content in cell walls (van der Weijde et al., 2013). However, as lignin provides mechanical support, stress response, and pathogen resistance to plants, decreased lignin contents can hamper plant stability and growth, and ultimately reduce biomass yield (Wang et al., 2016). Moreover, lignin itself represents an economically attractive compound, as it is a source of aromatic molecules that can find applications in the production of phenolics, carbon fibers, dispersants, and bio-plastics (Isikgor and Becer, 2015). Therefore, strategies aimed at modifying the lignin properties that affect biomass digestibility without decreasing the total lignin content – such as altering ratios of lignin subunits, relocating lignin deposition, and modifying lignin backbone and linkages with polysaccharides – also represent valid breeding approaches to improve biomass quality (Verma and Dwivedi, 2014; Li et al., 2016; da Costa et al., 2019).

The lignin biosynthetic pathway is well characterized (Boerjan et al., 2003; Bonawitz and Chapple, 2010) and also highly conserved across vascular plants (Boerjan et al., 2003; Loque et al., 2015). These characteristics make candidate gene approaches particularly suitable to modify lignin properties, by identifying critical target genes within the lignin pathway, testing their effectiveness in model crops, and transferring successful approaches to other less-studied species, as orphan biomass perennials. For example, the relevance of down-regulating the Caffeoyl *O*-Methyltransferases (*COMTs*) to decrease lignin content and improve biomass digestibility for biofuel production has been firstly shown in model forage crops as maize, tall fescue, and alfalfa (Hisano et al., 2011). These results have pushed the successful reproduction of this approach in switchgrass (Fu et al., 2011), while gene cloning and *in silico* protein alignments led to the identification of *COMT* genes in miscanthus (Dwiyanti et al., 2014) and eucalyptus (Carocha et al., 2015), which can become future targets of genetic modification to improve biomass quality. According to this approach, the improvement of lignin properties in novel lignocellulosic perennials for MALs can benefit from the long list of successful modifications of lignin-related genes in model plants and staple biomass crops (see Liu et al., 2018 for a recent overview). Overall, these studies also highlight four important principles for lignin modification. First, lignin content is decreased more effectively when genes acting early in lignin biosynthesis are targeted (Chen and Dixon, 2007). Second, down-regulation of genes is more effective than knock-outs to minimize side-effects on plant growth (Xie and Peng, 2011). Third, pathway cross-talks and gene redundancy need to be carefully considered to exclude mechanisms that can limit the gains from targeted approaches (Torres et al., 2015b). Fourth, to reproduce successful transgenic approaches in novel biomass crops, the availability of transformation and regeneration protocols is critical (Clifton-Brown et al., 2018).

Besides genetic modification, large natural variation in lignin content and composition exists within biomass perennials promising for MALs, as miscanthus (Zhao et al., 2014), switchgrass (Yan et al., 2010), or willow and poplar (Kenney et al., 1990). Moreover, studies investigating the sources of such variability showed that it is typically highly heritable (Torres et al., 2015a; van der Weijde et al., 2017b), and therefore constitutes an important breeding resource. Alternatively, intra-specific crosses between accessions showing contrasting lignin profiles have been also successfully used to map lignin-related QTLs across a range of species (Thumma et al., 2010; Torres et al., 2015a; van der Weijde et al., 2017b), which open prospects for marker-assisted selection (MAS) of genotypes showing superior lignin profiles and high biomass degradability.

#### Cell Wall Polysaccharides

Cellulose and hemicelluloses represent attractive polymers for bio-based applications, as they constitute the bulk of energy contained into lignocellulosic biomass and can be used as platforms to produce several classes of valuable biochemicals (van der Weijde et al., 2013; Isikgor and Becer, 2015). Therefore, increasing the cellulose and hemicellulose content in cell walls and modifying their molecular properties that promote recalcitrance are also effective targets to improve biomass quality (Torres et al., 2015b). Cellulose recalcitrance depends on the degree of cellulose crystallinity and polymerization (Wang et al., 2016), and reducing these two parameters is critical to improve lignocellulose degradability (van der Weijde et al., 2013; Torres et al., 2015b; Allwright and Taylor, 2016). Conversely, hemicelluloses affect recalcitrance through their total content in cell walls and their degree of branching (Torres et al., 2015b; Wang et al., 2016). Specifically, as hemicelluloses cross-link cellulose and lignin, high hemicellulose content reduces cellulose crystallinity (Wang et al., 2016). At the same time, low xylan branching ensures an easy separation of hemicelluloses, cellulose and lignin during saccharification (Torres et al., 2015b).

The molecular alteration of cell wall polysaccharides is challenged by our limited knowledge on cellulose and hemicellulose biosynthesis and regulation. Research in this field is still at the level of functional genetics in model species as Arabidopsis, while attempts of candidate gene studies in biomass crops remain few, mainly restricted to poplar and – to a less extent – maize (Wang et al., 2016). Several studies assessed the effect of modifying cellulose synthases (*CESAs*) on cellulose properties. For example, Joshi et al. (2011) decreased cellulose crystallinity in poplar wood by overexpressing a constitutive *CESA* (*PtdCESA8*). Alternatively, Harris et al. (2009) mutated the *AtCESA3*, which led to a 34% reduction of cellulose crystallinity and a 151% increase of biomass degradability. Besides *CESAs*, numerous other genes affect the molecular properties of cellulose and hemicelluloses. Glass et al. (2015) reduced cellulose crystallinity and increased biomass yield in Arabidopsis by down-regulating a class C endoglucanase (*AtGH9C2*). Furthermore, Mortimer et al. (2010) mutated the two glycosyltransferases-coding loci *GUX1* and *GUX2* in Arabidopsis, achieving substitutions-free hemicelluloses and easily extractable xylans. Overall, these studies highlight promising targets to improve cell wall polysaccharides, for which homologs could be searched in biomass crops and considered for genetic modification or screening of allelic diversity for conventional breeding programs (Torres et al., 2015b). However, in order to better predict/avoid the (negative) pleiotropic side-effects that are often encountered in these studies, it is critical to develop a better, systemic, understanding of the genetic complexity of cellulose and hemicellulose biosynthesis and regulation, and their interplay with other plant metabolic pathways. For example, along with the decreased cellulose crystallinity, the poplar transgenic lines produced by Joshi et al. (2011) displayed also a decreased cellulose content, increased lignin, and stunt growth, which are all unwanted traits for elite lines of perennial biomass crops.

As for lignin, variability in the composition and structure of cell wall polysaccharides across species constitutes an important breeding resource. In this regard, Harris and Debolt (2008) reported large variation in cellulose crystallinity across a set of 35 plant species, while Vandenbrink et al. (2012) and Porth et al. (2013) found considerable intra-specific variation in sorghum and poplar association panels, respectively. Alternatively, Torres et al. (2013) found variable hemicellulose composition and substitution patterns in a maize doubled-haploid population, which correlated with differences in biomass digestibility. To conclude, when molecular markers are available or can be easily developed, variability can also be used for association mapping, leading to the identification of genomic regions associated with high biomass quality, as performed by Torres et al. (2015a) in maize or van der Weijde et al. (2017b) in miscanthus.

### Biomass Yield and Quality Stability Under Abiotic Stresses

Abiotic stresses as water surplus or deficit, extreme temperatures, and soil salinity are common constraints to agriculture on MALs, and are expected to intensify as a result of climate change (Jones et al., 2015; Quinn et al., 2015). These stresses discourage the cultivation of MALs, as they can hamper crop growth, reduce biomass yield and quality, and ultimately hinder a stable biomass supply, which would volatilize prices of raw biomass and bio-based commodities. Developing genotypes that show stability of biomass yield and quality under adverse abiotic conditions is thus pivotal to successfully allocate worldwide MALs to biomass production. Specifically, since abiotic stresses often occur in combination (e.g., heat and drought) or succession (e.g., waterlogging followed by drought) (Mickelbart et al., 2015), resistant varieties should possibly combine different sources of resistance to withstand multiple stresses at once.

#### Suboptimal Water Availability

Water shortage inhibits cellular expansion, hydration and photosynthesis, with negative impacts on plant germination, establishment, growth, nutrient assimilation and transport (Quinn et al., 2015), and ultimately biomass yield (Emerson et al., 2014). Flooding determines instead stomata closure and uptake of toxic compounds released by anaerobic microorganisms in anoxic soils, which inhibit nutrient transport and photosynthesis, damaging plant growth and yield (Quinn et al., 2015). Drought and waterlogging are major causes of yearly yield losses worldwide and are acquiring more-than-ever importance as a consequence of extreme atmospheric events in a changing climate (Jones et al., 2015). Moreover, common MALs characteristics as steep slopes, high erodibility, or poor drainage amplify the occurrence and effects of these stresses (Quinn et al., 2015), calling for crops able to maintain normal metabolism, growth, and yield under drought and flooding.

**FIGURE 2:**
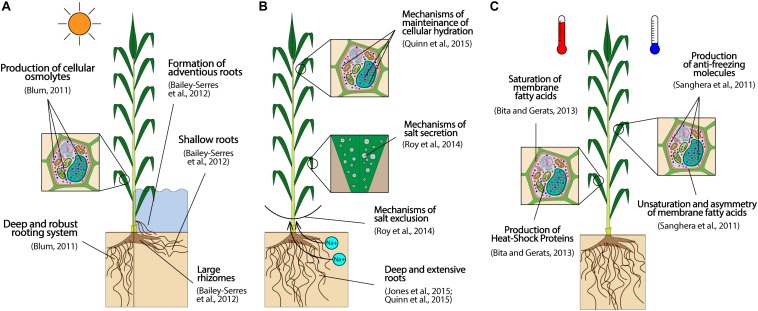
Preeminent target traits to equip perennial lignocellulosic biomass crops with effective resistance to common abiotic stresses of MALs: drought (**A**, left), flooding (**A**, right), salinity **(B)**, and extremely warm (**C**, left) or cold (**C**, right) temperatures. The traits reported represent an ideotype to guide breeding activities, and the effective magnitudes of improvement attainable with respect to each trait can vary extensively depending on the species object of a breeding program.

Several plant traits can be targeted to develop drought- and flood- tolerant crops, as visually summarized in Figure 2A. For drought, deep and robust roots, able to penetrate harsh soils, are important characters to reach deep water in dry areas (Blum, 2011). Moreover, the ability of accumulating cellular osmolites to avoid dehydration (osmotic adjustment) is also important, particularly at the seedling stage when roots are still underdeveloped (Blum, 2011), and can be improved by selecting against leaf rolling and in favor of green canopy under drought, which are two easily scorable traits (Amelework et al., 2015). For flood tolerance, a shallow root system, with a thick root epidermis, well-developed aerenchymatous tissues, and adventitious roots are critical traits as they facilitate aeration (Bailey-Serres et al., 2012). Moreover, large rhizomes are also favorable to provide starch to sustain optimal growth under prolonged flooding (Bailey-Serres et al., 2012).

Natural variation in tolerance to suboptimal water conditions is an important breeding resource, especially considering the high polygenicity of this trait (Mickelbart et al., 2015), and the difficulty of phenotyping roots in field conditions (see section “Efficiency of carbon partitioning into vegetative tissues”). Different WUEs between species are a first important variable trait, given the direct correlation between WUE and drought tolerance (Allwright and Taylor, 2016). In this regard, as previously discussed (see section “Efficiency of light conversion into biomass”), C4 crops display overall higher WUEs than C3 species, and are thus best options on dry MALs. In addition, intra-specific variation in the capacity of undergoing drought or floods without substantial yield penalties has been observed across several biomass perennials. For example, Barney et al. (2009) showed that upland and lowland switchgrass ecotypes display significantly different levels of drought tolerance. This observation is relevant, as it could enable to simultaneously improve drought tolerance and other traits displaying analogous latitudinal variation, such as flowering time, biomass yield, and cold resistance (Clifton-Brown et al., 2018). In miscanthus, van der Weijde et al. (2017a) have found large variation in the capacity of 50 accessions to produce biomass under drought, as their drought-constrained yields amounted to 30–110% of the yields under control conditions. The study assessed also biomass degradability under drought, demonstrating that it increased across all the 50 accessions, irrespectively of the level of drought tolerance, probably through an increase of the hemicellulose/cellulose ratio in cell walls (van der Weijde et al., 2017a). This is a remarkable finding, as it potentially allows to develop genotypes that are not only drought-tolerant, but also capable of turning the drought burden into an advantage for biomass quality. Finally, in black locust, Zhang et al. (2010) observed that tetraploid accessions withstand better drought than diploid genotypes, an information that can be useful at the moment of choosing superior lines to be used as parents in breeding programs (even if polyploidy can complicate breeding).

A better understanding of the genetics underlying drought and flood tolerance paves also the way to improve these traits through candidate gene approaches. Transcription factors (TFs) related to hormone metabolism are consistently accumulated under both drought and flooding across a range of plants (Yin et al., 2014; Mickelbart et al., 2015), and appear thus relevant to mine candidate genes. For example, Yang et al. (2015) and Zhao et al. (2016) enhanced drought, cold, and salt tolerance in Arabidopsis by ectopically overexpressing two NAC TFs from *Miscanthus lutarioriparius* (*MlNAC5*, *MlNAC9*) involved in the ABA-dependent signaling pathway. These results highlight the relevance of these genes to equip Miscanthus (and possibly other perennial grasses) with tolerance to multiple abiotic stresses, and their validity should be now investigated in target biomass crops. Alternatively, ERF-VIIs are TFs upregulated upon flooding across several plants (Mickelbart et al., 2015). They include the rice *SUB1A* gene, which provides flood tolerance to rice by quitting growth upon flooding, limiting resource dispersal (Xu et al., 2006). Manipulating the expression of ERF-VII TFs could thus be key to provide flood tolerance (Mickelbart et al., 2015), and deserves deeper investigation, especially in biomass crops. In fact, even if proper flood tolerance has not yet been improved through this approach (Peña-Castro et al., 2011), the expression of *OsSUB1A* in Arabidopsis not only led to flowering inhibition (mimicking the quiescence survival strategy observed in rice) (Peña-Castro et al., 2011), but also improved the saccharification efficiency of the biomass (Nunez-Lopez et al., 2015). Therefore, possibilities to improve both flood tolerance and biomass quality by manipulating ERF-VIIs could be furtherly explored. More in general, the interplay between cell wall quality and tolerance to abiotic stresses could be key to breed biomass crops, as cell wall composition and expression of cell wall genes are deeply changed under abiotic stresses, including drought and flooding (Le Gall et al., 2015; Houston et al., 2016). Unraveling genes acting upstream of both cell wall changes and induction of mechanisms of abiotic stress tolerance could thus represent a promising strategy to breed for both these traits in parallel.

#### Extreme Temperatures

Freezing (<−1°C), chilling (0–18°C), or high (>35–45°C, depending on species sensitivity) temperatures can physiologically and physically damage plants, penalizing biomass yield (Quinn et al., 2015). Cold temperatures reduce germination rates, growth, and tillering (Yadav, 2010), which delay or hamper biomass production (Jones et al., 2015). Moreover, cell wall composition is also affected by frost, probably with negative effects on biomass recalcitrance, as lignin content is typically increased (Le Gall et al., 2015). Heat stress shortens the growing season by accelerating feedstock maturation and inhibits photosynthesis, causing sugar catabolism (Quinn et al., 2015). Thus, both biomass yield (Bita and Gerats, 2013) and quality (Ananda et al., 2011) are penalized.

Tolerance to extreme temperatures starts by cultivating crops adapted to the temperature ranges of a target area. In fact, while temperate species – like many C3 crops (Jones et al., 2015) – are capable of cold acclimation to withstand chilling temperatures without impacts on crop performance, tropical and subtropical plants typically lack of such mechanisms, showing sensitivity even to chilling conditions (Sanghera et al., 2011). Conversely, crops from tropical and subtropical regions – such as the majority of C4 species (Schuler et al., 2016) – withstand better heat stress (van der Weijde et al., 2013), and could thus be preferentially planted in warm environments, where could eventually display combined tolerance to heat and drought. Once crops are correctly allocated, plant breeding can enhance freezing and heat tolerance to species already adapted to cool or warm environments, respectively. To this aim, high unsaturation and asymmetry in membrane lipids (which lower the membrane freezing point), and production of protective molecules (antioxidants, chaperones, deposition of apoplastic sugar) are important traits to prevent cellular damages from freezing (Figure 2C, right) (Sanghera et al., 2011). Conversely, heat tolerance is enhanced by production of heat-shock proteins (HSPs), secondary metabolites to protect against oxidative damage, and membrane saturated fatty acids to increase membranes melting point (Figure 2C, left) (Bita and Gerats, 2013).

Because of the complex molecular and genetic nature of the traits just mentioned, selection of tolerant accessions within pools showing variability for cold and heat resistance represents an effective breeding strategy (Bita and Gerats, 2013; Jones et al., 2015). In this respect, measuring electrolyte leakage following freezing is a rapid screening method for frost tolerance (Jones et al., 2015). Vice versa, assessing morphological traits that reduce heat load [e.g., pubescent, vertically oriented, or light-colored leaves (Quinn et al., 2015)], as well as physiological characters as maintenance of photosynthesis, chlorophyll content, and stomatal conductance under heat stress (Bita and Gerats, 2013) allows quick screening of heat tolerance. Furthermore, variability for both cold and heat tolerance has been observed for several biomass perennials (Quinn et al., 2015). For example, the level of frost tolerance is variable across diverse miscanthus genotypes (Clifton-Brown and Lewandowski, 2000; Farrell et al., 2006), and cold-tolerance could be coupled with breeding for earlier germination, which is also variable in miscanthus (Clifton-Brown and Jones, 1997) and is a target trait to increase biomass yield (see section “Efficiency of light interception”).

Besides selection within intra-specific variation, sources of both heat and cold tolerance can be also introgressed through crosses with wild relatives evolved under stressed environments, or by transgenic modification. The first approach has been successfully applied in wheat (Mohammed et al., 2014), and could be used in biomass crops in which different species show different levels of tolerance to extreme temperatures, as poplar [*Populus euphratica* is tolerant to both heat and drought, and candidate genes and mechanisms underneath tolerance have been identified (Ferreira et al., 2006; Tang et al., 2013)] or sugarcane [the wild species *Saccharum spontaneum* shows higher cold tolerance than the most cold tolerant commercial varieties of sugarcane (Hale et al., 2014)]. The second approach has instead been successfully applied in eucalyptus, where the transgenic introgression of *CBF* genes [a class of TFs that promote cold acclimation across a range of plants (Mickelbart et al., 2015)] has greatly improved cold tolerance (Hinchee et al., 2011). As several genes involved in cold and heat tolerance are known across plants (see Sanghera et al., 2011 for a review), this approach could be reproduced also with other species and other targets.

#### Salinity

Soil salinity hampers plant growth as it reduces osmotic potential – which challenges water uptake and solute movement – and causes ion cytotoxicity as salt ions compete with potassium to occupy enzymatic active sites (Mickelbart et al., 2015; Quinn et al., 2015). In biomass crops, salinity consistently decreases biomass yield (Quinn et al., 2015) and remodels cell wall composition, which is thought to negatively affect biomass quality, as lignin content is typically increased (Le Gall et al., 2015).

Not all biomass crops are salt sensitive, and the allocation of tolerant species to salty MALs is a preeminent strategy to avoid salt-induced penalties to biomass production (Jones et al., 2015). In this regard, some herbaceous biomass perennials as giant reed or *Pennisetum purpureum* display striking levels of salt tolerance, comparable with halophytes (Quinn et al., 2015). Furthermore, biomass trees as *Eucalyptus camaldulensis*, several poplar and willow hybrids, and tetraploid *Robinia pseudacacia* accessions also show high tolerance to salinity (Quinn et al., 2015). Globally, all these species represents good options for salty MALs.

Crops can also be equipped with mechanisms of salt tolerance through plant breeding. Since salty and dry soils are functionally similar, plant traits benefiting drought tolerance are also effective to improve salt tolerance (Quinn et al., 2015). Specifically, deep and large root systems and the capacity of maintaining cellular hydration appear particularly critical characters, as shown in Figure 2B. These traits allow plants to search for water and nutrients in deep soil layers less affected by salinity, and to keep water-to-salt ratios in cells at acceptable levels (Jones et al., 2015; Quinn et al., 2015). In addition, mechanisms of salt exclusion from shoots, and secretion of excess salt uptake are other effective characters to provide salt tolerance (Figure 2B) (Roy et al., 2014), and can be effectively phenotyped by screening ion levels in plant tissues (i.e., total Na^+^ and Cl^–^ contents and K^+^/Na^+^ ratio) (Chen, 2018). For example, Chen (2018) has successfully applied this method to screen levels of salt tolerance across 70 miscanthus genotypes, revealing a relatively high degree of genetic diversity in the ability of withstanding salinity among the accessions. The study has specifically showed the presence of genotypes capable of excluding salt ions from shoots, preventing leaf senescence, and sustaining biomass production on salty soils (Chen, 2018). These genotypes could be used as parents in future miscanthus breeding programs (Chen, 2018). Besides miscanthus, genetic variability for biomass production under salinity has been observed also in other biomass perennials, as switchgrass (Liu et al., 2014), black locust (Wang et al., 2013), or poplar (Sixto et al., 2005), and could be used for breeding. However, the assessment of the capacity of maintaining biomass quality under salinity – in addition to biomass yield – is often skipped by studies investigating salt tolerance. This is a major shortcoming that can lead to negative selection biases (i.e., selection of salt tolerant genotypes that eventually turn out to be highly recalcitrant to biomass processing), as studies in miscanthus have shown that best-performing accessions for salt tolerance do not coincide with genotypes showing highest biomass quality under salt stress (Chen, 2018).

## Tools and Strategies to Advance Promising Lignocellulosic Perennial Crops for MALs

### Available Breeding Tools

Until now, only a limited set of lignocellulosic biomass crops promising for MALs have undergone breeding (Allwright and Taylor, 2016), while the majority lies in a state close to (selected) wild germplasm (see Table 1). This small set of species includes miscanthus, switchgrass, willow, poplar, and eucalyptus, whose improvement history dates back to the second half of the twentieth century (Grattapaglia and Kirst, 2008; Allwright and Taylor, 2016; Clifton-Brown et al., 2018). All these species are outcrossing, and until early 2000s their genetic improvement has entirely relied on phenotypic selection within breeding populations created through recurrent inter-mating of wild and advanced germplasm, or intra- and inter-specific hybridization (Grattapaglia and Kirst, 2008; Clifton-Brown et al., 2018). These methods allow to combine favorable traits from distant genetic pools into elite lines and to exploit heterosis (given that the genetic structure of target populations and heterotic compatibility are analyzed prior to design breeding schemes) (Acquaah, 2012). However, the release of commercial varieties takes long time (i.e., 11–26 years), which significantly delays the adoption into real agricultural contexts of the improvements achieved in breeding programs (Clifton-Brown et al., 2018). This is due to the long period (sometimes several years) that perennial crops often require to phenotypically express traits of interest, the long breeding cycles (especially in biomass trees), and the prolonged time to commercially upscale elite lines of highly heterozygous outcrossing species (Clifton-Brown et al., 2018).

Over the last decades, research investments have permitted to develop advanced tools to assist the improvement of these crops at all the breeding levels (Clifton-Brown et al., 2018). The first tools created were genetic maps which, together with the use of mapping populations segregating for a target character that is phenotypically divergent between the founder individuals, have enabled to map quantitative trait loci (QTLs) for growth, quality, and agronomic traits (see Table 1 for relevant references). Initially, QTL detection has exclusively relied on bi-parental crosses, but more recently multi-parental approaches to mine polymorphisms linked to loci of interest – which allow to enhance the allelic diversity and variety of genetic backgrounds included in a study – have begun to be applied also in biomass crops (Mandrou et al., 2014). The availability of markers and QTLs has in turn enabled marker-assisted selection (MAS) (Clifton-Brown et al., 2018). MAS encompasses genotyping breeding material for the alleles harbored at marker loci associated with QTLs of interest, and selecting accessions carrying positive alleles at those marker loci. Therefore, selection can take place already at early developmental stages, even before a trait is phenotypically expressed, which can significantly accelerate breeding gains. For example, Thavamanikumar et al. (2018) applied MAS to improve wood density, pulp yield, and total plant growth in eucalyptus, showing that it can reduce by 50% the duration of breeding cycles (from 10–15 to 5–7 years), while breeding gains can be achieved at a 2–3 fold higher rate than by conventional selection.

The drop of genotyping costs brought by genotyping-by-sequencing (GBS) technologies, coupled with the release of the genome sequences of all the species referred in here [Tuskan et al. (2006) (poplar), Myburg et al. (2014) (eucalyptus), Dai et al. (2014) (willow)^1^, (miscanthus, switchgrass)] has permitted the development of dense arrays of single nucleotide polymorphisms (SNPs) covering the whole genome, which have in turn paved the way to genome-wide association studies (GWAS) and genomic selection (GS) schemes (Allwright and Taylor, 2016; Clifton-Brown et al., 2018). GWAS is a powerful tool to detect marker-traits associations using genotypic collections inclusive of long recombination histories, which promises to achieve deep mapping resolutions, and save the time needed to set up experimental populations for QTL mapping (Bernardo, 2016). Because of their high genetic diversity, undomesticated status, and generally fast linkage disequilibrium (LD) decay, lignocellulosic crops – especially biomass trees – appear ideal for GWAS (Du et al., 2018), and several studies have thus used this approach to reveal loci underlying biomass-related traits. For example, GWAS has been used in poplar to detect several marker-trait associations for quality characters as lignin content and composition (Porth et al., 2013), as well as for phenology traits as canopy duration or flowering date (McKown et al., 2014). Despite GWAS promises, Fahrenkrog et al. (2017) have pointed out how rare allele variants, whose detection can be quite often missed by GWAS analyses (Bernardo, 2016), can be particularly relevant to explain genetic variation for bioenergy traits as cell wall composition. Therefore, good experimental designs (e.g., adequate sample size and geographical sampling of accessions to give a balanced representation of the variability for a trait of interest in the panel used; Brachi et al., 2011) are pivotal to successfully perform a GWAS (Du et al., 2018). As for QTL mapping, GWAS results can be directly used for MAS (Allwright and Taylor, 2016). However, genome-wide marker allelic effects from GWAS analyses can also be used to calculate breeding values for every individual plant in a breeding population, which is the concept standing behind genomic selection (GS) (Heffner et al., 2009). GS is particularly suited for crops showing large phenotypic and genetic variability, as miscanthus (Allwright and Taylor, 2016), where the feasibility of applying this strategy has started to be explored (Davey et al., 2017). To conclude, marker arrays are also useful to screen the genetic diversity of novel germplasm collections, which is a common need of pre-breeding research in orphan crops (Clifton-Brown et al., 2018). Diversity screenings can be informative to establish the geographical origin and the relatedness with other plant material, which are important information to take breeding decisions (Narasimhamoorthy et al., 2008; Lu et al., 2013).

Research on miscanthus, switchgrass, poplar, willow and eucalyptus has also aimed at developing transformation protocols to insert genes underlying traits hardly found in extant accessions. Clifton-Brown et al. (2018) and Kendurkar and Rangaswamy (2018) have recently reviewed the progress achieved in this field across these five crops, highlighting that stable protocols are available for switchgrass, miscanthus, and poplar, while willow and eucalyptus can display recalcitrance to transformation. In addition, several studies demonstrated the efficacy of genetic modification to improve traits for which critical candidate genes are known, as discussed in section “Target traits and genetic resources to tailor novel biomass crops to MALs.” Overall, the public acceptance of genetic modification for biomass crops grown for bio-based applications could be higher than that for food crops (van der Weijde et al., 2013). However, when transgenic lines of outcrossing species were effectively cultivated, measures of gene confinement should be designed, as (trans)gene flow to relative wild species could be an issue (Clifton-Brown et al., 2018).

To conclude, fast and cost-effective phenotyping is also an asset to improve understudied crops, for which screening large germplasm collections is fundamental to evaluate variability for breeding programs (Clifton-Brown et al., 2018). Recent advances in high-throughput phenotyping open promising prospects in this regard. Fernandez et al. (2017) developed a robotic workstation that can be used to phenotype yield-related traits in tall herbaceous biomass crops as sorghum. The system has been successfully used to phenotype stem diameter and plant height in a GWAS sorghum panel, and the data collected allowed the detection of known QTLs for these traits, demonstrating the efficacy of this platform (Fernandez et al., 2017). Near-infrared spectroscopy (NIRS) technologies offer instead a viable option for high-throughput phenotyping of cell wall compositional traits, and robust protocols for their application have been recently developed and successfully used to phenotype a mapping population of miscanthus (van der Weijde et al., 2017b). Finally, thermal aerial imaging constitutes a high-throughput option to screen abiotic stress tolerance, and Ludovisi et al. (2017) have reported its successful application to phenotype the drought response in a large black poplar population consisting of 4603 individuals (503 genotypes). These examples clearly highlight how novel phenotyping technologies can widen the scale and enhance the speed of breeding programs, and need to be considered when improving novel biomass crops.

### Prospects for the Improvement of Orphan Lignocellulosic Biomass Species

The tools above are effective for crop improvement, but they are available just for a handful of lignocellulosic species, and their *de novo* development for orphan crops would require time and adequate research investments [even if the drop of sequencing costs will soon allow association mapping and whole-genome sequencing also to novel crops (Allwright and Taylor, 2016)]. Conversely, we have seen that classical breeding alone is also time-consuming and not very effective. In this scenario, genetic tools to transfer genetic knowledge from model species to less-studied crops and to meaningfully coalesce genetic information on relevant traits across crops can be key to bridge the gap between advanced and orphan biomass species.

Translational genomics offer a possibility in this perspective, as it allows the identification of candidate genes underlying a trait of interest in a “target” organism based on its homolog(s) in a model species (Salentijn et al., 2007). Such candidate genes can then be targeted through genetic modification to obtain a desired phenotype (see section “Target traits and genetic resources to tailor novel biomass crops to MALs” for examples). This approach allowed the identification and modification of several of the candidate genes discussed in section 3, and is particularly powerful for plant clades that share high levels of genome synteny between members and include model bioenergy crops (van der Weijde et al., 2013), as grasses (Bennetzen and Freeling, 1997; Carpita and McCann, 2008). To facilitate translational genomics, several tools have been developed over the years, in the form of both genomic databases [e.g., PlantGDB^2^ (Dong et al., 2004), Plaza 4.0 (Van Bel et al., 2017)^3^, or other grass-specific databases reviewed by van der Weijde et al. (2013)] and platforms specifically designed for orphan crops lacking of a sequenced genome, but for which transcriptomes can be developed (e.g., Orphan Crops Browser; Kamei et al., 2016)^4^.

Most of the traits discussed in section “Target traits and genetic resources to tailor novel biomass crops to MALs” are highly quantitative, and the available knowledge on the genetics underlying them in model species is typically in the form of QTLs with no validated or known candidate genes (Barrière et al., 2007). In these cases, tools to meaningfully coalesce the information on relevant QTLs between species and make it inter-applicable in a way immediately usable in MAS or GS contexts would be very useful. Combining meta-QTL analysis approaches (Goffinet and Gerber, 2000) extended beyond species boundaries with the development of “universal markers” that are present across species but can assay intra-specific diversity for traits of interest (Ranade and Yadav, 2014) could offer promising possibilities in this direction. Such universal markers could effectively allow to project known QTLs to breeding material not included in the original panels used for QTL mapping, or even to possibly other (orphan) species, on which MAS based on universal markers could take place, without the need of *de novo* producing species-specific knowledge. The extensive occurrence of common genetic factors underlying complex biomass-related traits across evolutionary distant plant species (as exemplified for cell wall recalcitrance in section “Target traits and genetic resources to tailor novel biomass crops to MALs”) promises success from the application of the approach just described. However, research is needed to define to which extent common genetic determinants of traits of interest show positional conservation of their genomic organization to allow inter-species projection of QTLs and universal markers. In this direction, novel high-throughput tools to assess overall syntenic relationships between genetic elements underlying critical traits across large sets of plant genomes not even always displaying high levels of collinearity can offer promising prospects (Zhao et al., 2017; Zhao and Schranz, 2017).

Universal markers as just defined would represent a very useful tool to overcome the condition of orphan crops in which several promising species for marginal lands lay. However, these tools – as well as all the other genomic, molecular, and biotechnological approaches discussed in this review – do not represent *per se* a “finish line” in breeding novel promising perennial crops for marginal lands. Their effectiveness will ultimately depend by the specific ways in which breeders will integrate these tools in well-planned and modern “knowledge-based” breeding programs. Specifically, such programs will continue to largely rely on pre-breeding activities (i.e., germplasm development, dissection of the genetics underlying the traits discussed in section “Target traits and genetic resources to tailor novel biomass crops to MALs,” and development of markers), conventional crossing of promising accessions and selection within progenies, as well as ongoing schemes of population improvement through recurrent selection (especially in open-pollinated species) (Clifton-Brown et al., 2018). Nevertheless, the tools discussed in this review will allow to speed up major steps of such programs (from the genetic characterization of breeding material and the dissection of the genetic determinants of target traits, to phenotyping, marker development, or the targeting of critical genes by genetic modification), as well as precisely guide breeding activities in crops that so far have been poorly studied. This aspects will ultimately be key to ensure that novel lignocellulosic perennials will be advanced at a sufficient level for commercialization in a reasonable time, which is currently the major priority for using MALs to provide biomass for a bio-based economy.

## Concluding Remarks

MALs have great potential to sustainably supply a large proportion of the biomass needed to fuel a global bio-based economy. However, the lack of crop varieties that can couple sustainability of biomass production with optimal biomass yield and quality to ensure profitable cultivation of MALs and cost-effective biomass conversion into bio-based commodities currently impedes to realize this vision. We firmly believe that plant breeding will be key to break through this impasse, and in this article we have dissected the problem of biomass provision using MALs from a plant breeding perspective. What emerges is that great progress has been made over the years in understanding the genetics underlying biomass traits. Moreover, the development of tools to study these aspects on larger scales and through quicker approaches will expand this knowledge in the future. Progress is, however, uneven among crops. While a few model species can count on an array of breeding tools and genetic knowledge to support their improvement but are unsuitable for sustainable cultivation on MALs, a wide range of locally adapted crops cannot be readily improved being paradoxically orphan in the genomic era. Therefore, our ability of creating tools to effectively transfer and coalesce the genetic knowledge on traits of interest across crops and to integrate such tools into modern, “knowledge-based” breeding programs will ultimately represent a key factor to enable the development of biomass crops tailored to the needs of MALs and to a bio-based economy.

## Author Contributions

FP wrote this review, with input from LT. LT corrected the manuscript. FP and LT approved the final manuscript.

## Conflict of Interest

The authors declare that the research was conducted in the absence of any commercial or financial relationships that could be construed as a potential conflict of interest.

## Footnotes

1 http://phytozome.jgi.doe.gov/

2 www.plantgdb.org

3 https://bioinformatics.psb.ugent.be/plaza/

4 http://www.bioinformatics.nl/denovobrowser/db/species/index
